# From Brain to Blood to Skin: Compartment Biology, Interstitial Fluid Dynamics, and Microneedle Sampling of Neurological Biomarkers

**DOI:** 10.3390/brainsci16101057

**Published:** 2026-09-30

**Authors:** Maria Y. Y. Shiu, Melissa Cote, Nigel McLeish, Jun Han Bae, Shawn G. Rhind

**Affiliations:** 1 Toronto Research Centre, Defence Research and Development Canada (DRDC), 1133 Sheppard Avenue West, Toronto, ON M3K 2C9, Canada; maria.shiu@ecn.forces.gc.ca; 2 Canadian Forces Environmental Medicine Establishment, 1133 Sheppard Avenue West, Toronto, ON M3K 2C9, Canada; mcote009@gmail.com (M.C.); nigel.mcleish@ecn.forces.gc.ca (N.M.); junhan.bae@forces.gc.ca (J.H.B.); 3 Faculty of Kinesiology and Physical Education, University of Toronto, 55 Harbord Street, Toronto, ON M5S 2W6, Canada

**Keywords:** neuroinjury biomarkers, neuroproteomics, cerebrospinal fluid, glymphatic system, blood–brain barrier, dermal interstitial fluid, microneedles, GFAP, neurofilament light chain, traumatic brain injury

## Abstract

Proteins released after brain injury can be measured in blood, but a blood result does not directly measure the concentration at the site of injury. Between brain tissue and a peripheral sample, proteins may enter brain interstitial fluid (ISF), exchange with cerebrospinal fluid (CSF), leave through several drainage or barrier pathways, and undergo dilution and clearance. This narrative review examines those steps for glial fibrillary acidic protein (GFAP), ubiquitin C-terminal hydrolase-L1 (UCH-L1), neurofilament light chain (NfL), S100B, neuron-specific enolase (NSE), tau proteoforms, and amyloid-beta. It also evaluates whether microneedle sampling of dermal ISF (dISF) could support repeated, low-burden measurement. The evidence indicates that gradients and time lags differ by analyte; a single blood-to-skin conversion is unlikely to work. Human dISF studies demonstrate broad molecular overlap with plasma, including similar N-glycan profiles despite lower total protein, and a human aptamer-patch trial demonstrates frequent drug monitoring. These findings do not yet validate a neurological protein assay in skin: no paired clinical study has established quantitative dISF concordance for GFAP, UCH-L1, NfL, tau, or amyloid-beta. The immediate priorities are analyte-specific recovery testing, paired blood–dISF time courses, paired CSF measurements where clinically feasible, and careful accounting for local skin and device effects.

## 1. Introduction

The interstitium is a body-wide, multiscale network of fluid-filled extracellular spaces that surrounds cells and microvessels and extends through the dermis, fascia, vascular adventitia, perineurium, and connective tissues of organs. Rather than being a passive space, it is a dynamic exchange and signaling compartment linking tissue cells with the blood circulation and lymphatic drainage. Water, solutes, and proteins enter local interstitial fluid (ISF) through capillary exchange and cellular release, and their subsequent movement is shaped by diffusion, pressure gradients, extracellular-matrix interactions, local metabolism, and lymphatic or vascular clearance. Although interstitial spaces exhibit anatomical continuity across many tissue planes, they do not equilibrate rapidly or uniformly; regional structure and fluid dynamics can create analyte-specific gradients and time lags. Within the central nervous system (CNS), this broader transport framework encompasses specialized exchange among brain ISF, cerebrospinal fluid (CSF), perivascular pathways, the cerebral vasculature, and meningeal and cervical lymphatic drainage. The interstitium is therefore central to understanding how neurological biomarkers travel from brain tissue to blood and, ultimately, dermal ISF [1,2].

Within this framework, protein biomarkers of neurological injury, whether measured individually or through proteomic methods, can provide accessible indicators of CNS injury in living individuals. In acute traumatic brain injury (TBI), selected blood tests already inform clinical evaluation, while serial measurements may help characterize secondary injury and recovery [3,4]. The clinical and public-health need is considerable: TBI affects an estimated 69 million people worldwide each year [5], and neurological disorders, including Alzheimer’s disease (AD), impose a substantial global burden [6]. However, interpreting a concentration measured in blood requires consideration not only of cellular release but also of the pathways through which the protein leaves the brain and reaches the systemic circulation.

**Figure 1:**
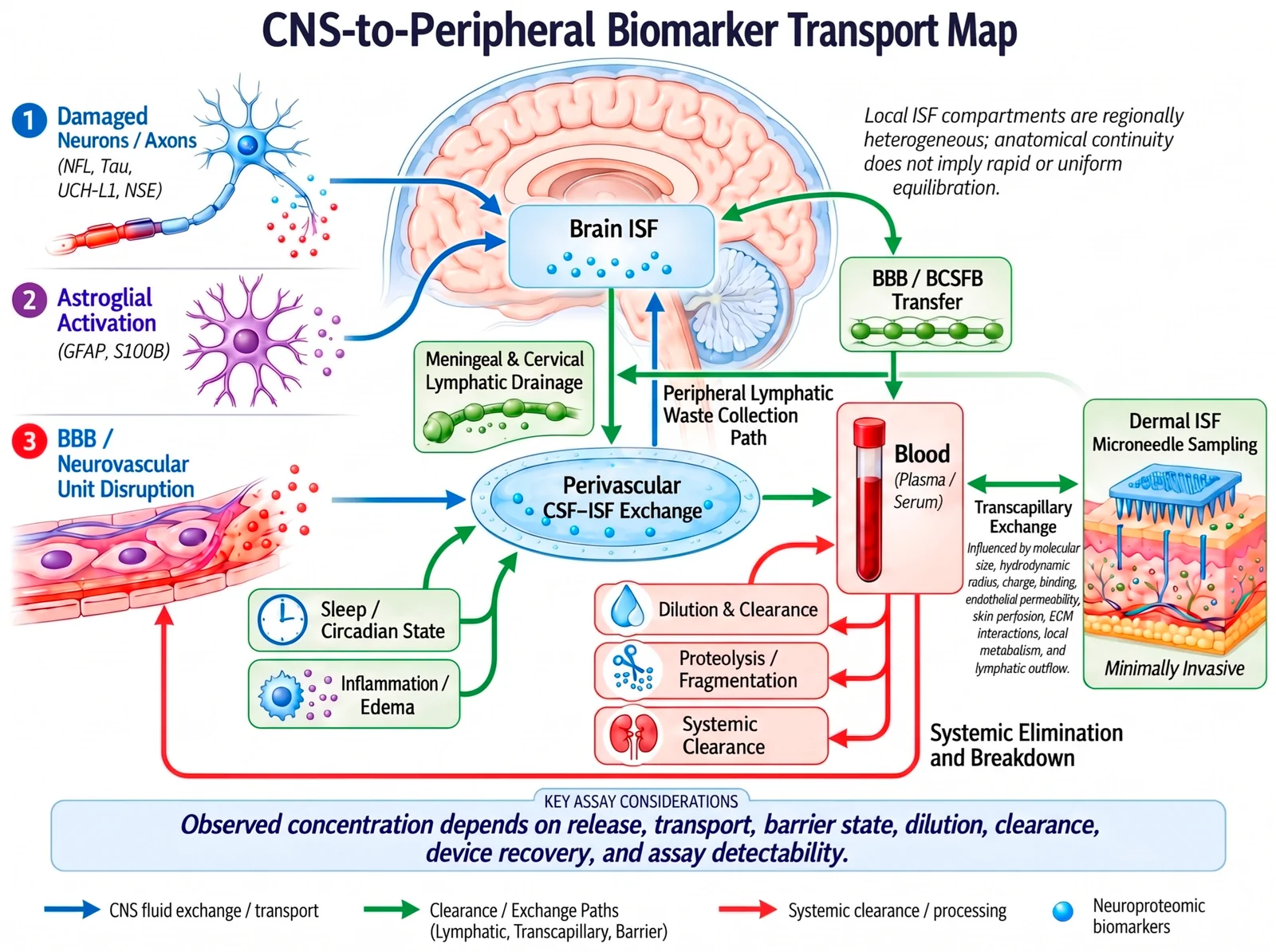
Conceptual model of central nervous system (CNS)-to-peripheral transport of neurological protein biomarkers. Following neurological injury or disease-related cellular perturbation, proteins released from damaged neurons and axons—including neurofilament light chain (NfL), tau, ubiquitin C-terminal hydrolase-L1 (UCH-L1), and neuron-specific enolase (NSE)—and from activated or injured astroglia, including glial fibrillary acidic protein (GFAP) and S100 calcium-binding protein B (S100B), enter brain interstitial fluid (ISF). Biomarkers may subsequently exchange between brain ISF and cerebrospinal fluid (CSF) and reach the systemic circulation through partially overlapping pathways, including perivascular CSF–ISF exchange described by the glymphatic model, meningeal and cervical lymphatic drainage, and transfer across blood–brain barrier (BBB) or blood–CSF barrier (BCSFB) interfaces. The relative contribution of these pathways is analyte- and context-dependent and remains incompletely quantified in humans. Transport may be influenced by sleep and circadian state, inflammation, edema, neurovascular function, barrier integrity, and injury severity. Once in blood, measured concentrations are further shaped by dilution, protein binding, proteolysis or fragmentation, and renal, hepatic, or other systemic clearance. Circulating proteins may exchange across the dermal microvasculature with dermal ISF, where movement and recovery are influenced by molecular size and charge, binding, endothelial permeability, skin perfusion, extracellular-matrix interactions, local metabolism, lymphatic outflow, and device characteristics. Microneedle-based devices may enable minimally invasive serial sampling of this compartment. Because local ISF compartments are regionally heterogeneous, anatomical continuity does not imply rapid or uniform equilibration among brain ISF, CSF, blood, and dermal ISF. Accordingly, the concentration measured in any compartment represents the combined influence of cellular release, transport, barrier state, dilution, molecular processing, clearance, sampling recovery, and assay detectability. Arrows indicate plausible and potentially concurrent biological pathways and do not establish fixed directionality, relative flux, or quantitative contribution. Abbreviations: BBB, blood–brain barrier; BCSFB, blood–CSF barrier; CNS, central nervous system; CSF, cerebrospinal fluid; ECM, extracellular matrix; GFAP, glial fibrillary acidic protein; ISF, interstitial fluid; NfL, neurofilament light chain; NSE, neuron-specific enolase; NVU, neurovascular unit; S100B, S100 calcium-binding protein B; UCH-L1, ubiquitin C-terminal hydrolase-L1.

For brain-derived biomarkers, transport begins with cellular release into brain ISF. Proteins may then exchange with CSF and reach peripheral compartments through partially overlapping perivascular, meningeal lymphatic, and other outflow pathways. Transfer across the blood–brain barrier (BBB), formed largely by brain microvascular endothelium, or the blood–CSF barrier (BCSFB), primarily at the choroid plexus, may also contribute. Barrier permeability can change after injury and influence blood concentrations, but it cannot explain every peripheral signal. The glymphatic model describes perivascular CSF–ISF exchange associated with aquaporin-4 (AQP4) localization at astrocytic endfeet [7], while meningeal lymphatics provide an additional connection to peripheral drainage [8,9,10]. The relative contribution of these routes for any particular protein remains debated [11,12,13,14,15]. Accordingly, we use the concept of CNS interstitial fluidopathy [16] to examine how altered fluid handling could complicate biomarker interpretation. Experimental studies support the movement of injury-associated proteins through some of these pathways [17,18] but do not yet quantify the contribution of each route in humans. Figure 1 follows these compartments from neural release to blood and skin.

The proteins considered here do not all report the same injury process. Glial fibrillary acidic protein (GFAP) and S100 calcium-binding protein B (S100B) are associated with astroglia, although S100B also has substantial peripheral sources. Ubiquitin C-terminal hydrolase-L1 (UCH-L1) is enriched in neuronal cell bodies, neuron-specific enolase (NSE) is a neuronal enzyme with important extracranial and erythrocyte sources, and neurofilament light chain (NfL) reflects the axonal cytoskeleton. Tau occurs in several assay-defined forms, and amyloid-beta (Aβ) illustrates the importance of production and clearance. We examine whether any of them can be recovered reproducibly from dermal ISF (dISF), linked to paired blood or, where clinically indicated, CSF, and sampled often enough to follow their kinetics. Our working hypothesis is that this relationship will differ among analytes, sampling devices, and physiological states. Microneedles may reduce the burden of repeated blood collection, but dISF measurement remains an investigational complement to established blood testing, cerebral microdialysis, and clinically indicated CSF sampling.

## 2. Materials and Methods

We conducted a narrative review of protein biomarkers of neurological injury, CNS compartment physiology, and minimally invasive dISF sampling. PubMed/MEDLINE, Scopus, and Web of Science were searched for English-language peer-reviewed publications from 1 January 2000 to 23 August 2026; earlier landmark studies were retained where necessary. Search concepts covered GFAP, UCH-L1, NfL, S100B, NSE, tau, and amyloid-beta; brain ISF, CSF, glymphatic and meningeal lymphatic transport, BBB and BCSFB; and microneedles, hydrogel, hollow devices, wearable sensors, proteomics, and glycomics. We also followed citations in relevant primary studies and reviews. We selected studies that informed compartment biology, analyte kinetics, sampling recovery, analytical performance, paired-matrix relationships, or potential clinical and operational applications. Paired human investigations and primary analytical validation were prioritized; animal and engineering studies were used to examine mechanism and feasibility. Because populations, assays, matrices, and devices varied substantially, we synthesized findings qualitatively and distinguished clinical evidence from human feasibility, analytical, and preclinical evidence.

## 3. Compartment Biology: What Each Sample Represents

The sampled compartment determines what a protein measurement can tell us. Brain ISF is local to neurons, glia, and the neurovascular unit; CSF collects signals over a broader spatial scale but varies with turnover, sampling site, and exchange with brain ISF [19,20]. In blood, transport through drainage pathways or across the BBB and BCSFB is followed by processing, binding, dilution, and clearance [21,22]. dISF surrounds skin cells and exchanges with plasma across the dermal microvasculature. It is neither a direct sample of brain ISF nor a fixed dilution of plasma. Local perfusion, tissue metabolism, protein binding, molecular size, device design, and collection time all matter [23,24,25,26]. A paired study in five healthy volunteers reported roughly threefold less total protein in dISF than plasma but closely overlapping individual N-glycan profiles [27]. N-glycans are carbohydrate chains attached to proteins; similar aggregate patterns suggest that many glycoproteins can be sampled in both matrices, but they do not prove that the same low-abundance brain-derived protein has the same concentration or kinetics in each.

### 3.1. Brain Interstitial Fluid and Cerebral Microdialysis

Cerebral microdialysis samples extracellular solutes next to a probe implanted in the brain. Perfusate flows through the probe, and molecules in the surrounding brain ISF must cross its semipermeable membrane to enter the collected dialysate; the membrane cut-off, flow rate, and adsorption therefore determine how much of a given protein is recovered [28,29]. The extracellular space occupies approximately 20% of brain volume, and its geometry constrains diffusion [30,31]. This is a local sample, not a whole-brain concentration. In human TBI, cerebral microdialysis has revealed time-varying cytokine profiles [32,33]. In seven patients with severe TBI, paired microdialysate and CSF measurements of S100B, NSE, total tau, and phosphorylated tau showed markedly different temporal patterns among analytes and patients [34]. There is no basis in these data for a universal ISF-to-CSF conversion factor.

Alternative methods can recover some molecules that conventional microdialysis under-samples. In mice, paired microdialysis-based brain-ISF and CSF proteomics identified up to 625 and 4483 proteins, respectively; Gfap was among the regulated ISF candidates in an amyloid model [35]. A micro-invasive, membrane-free method collects fluid without the dialysis membrane described above and may improve recovery of larger proteins [36]. Related work identified region-specific ISF proteomes in the nucleus accumbens and substantia nigra [37], while a separate hippocampal method recovered extracellular vesicles for proteomic analysis in living mice [38]. These results expand the number and types of molecules detectable in experimental ISF samples. They do not establish the same recovery in humans or in dermal ISF.

Primary studies also define analyte-specific brain-ISF kinetics. In vivo microdialysis established the measurement and half-life of soluble amyloid-beta (Aβ) in awake mice [39]. In acutely brain-injured patients, serial brain-ISF Aβ dynamics tracked neurological status [40]; severe-TBI studies measured total tau and Aβ [41] and linked early extracellular tau elevations with reduced Aβ and adverse outcomes [42]. In 11 patients with suspected normal-pressure hydrocephalus, 24 h microdialysis detected Aβ42, total tau, and p-tau181 and showed distinct early tau kinetics [43]. Together with the broader TBI literature [44], these data position brain ISF as an analyte- and site-specific mechanistic anchor, provided that recovery and sampling context are made explicit.

### 3.2. Cerebrospinal Fluid and Neurofluid Dynamics

CSF is a proximal biofluid that reflects dynamic exchange with brain ISF through perivascular and interstitial pathways. CSF biomarker concentrations reflect not only CNS injury and disease processes but also transport and clearance dynamics within the neurofluid system [19,20]. The glymphatic hypothesis frames CSF–ISF exchange as a mass-transport system influenced by perivascular flow and AQP4-dependent water movement through astrocytic endfeet [7,8]. Tau and Aβ are detectable in human brain ISF, but their movement among ISF, CSF, and blood is proteoform-, barrier-, and clearance-dependent [41,42,43]. Impaired neurofluid dynamics, termed ‘CNS interstitial fluidopathy’, is increasingly recognized across disorders including TBI, AD, Parkinson’s disease, stroke, and multiple sclerosis [11,13,16,45]. When CSF is available, the QAlb (CSF/serum albumin quotient) provides a practical index of barrier permeability to contextualize CSF–blood biomarker discordance [46]. However, the biological specificity of glymphatic-related imaging proxies remains debated: DTI-ALPS reflects localized directional diffusivity shaped by white matter microstructure rather than a direct measure of whole-brain fluid transport and is increasingly reframed as a composite, structure-dependent marker rather than a validated glymphatic flow index [47,48]. Illustrating this uncertainty, a pilot study of anti-amyloid immunotherapy (lecanemab) found that ALPS index and choroid plexus volume changes did not consistently track concurrent improvements in CSF amyloid and tau biomarkers over 12 months, suggesting these imaging proxies may be insufficient as stand-alone surrogate endpoints [49].

### 3.3. Blood and Dermal Interstitial Fluid

Blood is a distal compartment: protein signals reaching it have already been shaped by transport, dilution, binding, processing, and clearance [17,18,50,51]. CSF and blood concentrations may differ by orders of magnitude, with the gradient changing by analyte, assay, disease, and sampling time [52,53]. dISF exchanges with plasma through capillary filtration and diffusion, while lymphatic removal and local metabolism also affect its composition. Molecular mass matters, but hydrodynamic radius, molecular charge, conformation, binding, endothelial permeability, and skin perfusion may matter just as much [25,26,54,55]. Aptamer-sensor experiments show that protein binding can restrict access to dISF [56]. Extracellular-matrix binding also makes some dermal fluid and its proteins difficult to extract [57]. Dermal-ISF immunoglobulin profiles resemble those of plasma but contain relatively less IgM, consistent with size-dependent partitioning and possible local immune contributions [58].

For a protein moving from blood into skin, a useful first approximation is a plasma compartment, a permeable dermal microvasculature, and a dermal interstitial compartment with its own distribution volume and lymphatic outflow. The blood-to-dISF concentration difference drives exchange; the capillary permeability–surface-area product, vascular reflection and binding, effective interstitial volume, and local clearance determine how quickly the measured concentration approaches a new level. A larger hydrodynamic radius or greater protein binding can restrict capillary passage and lengthen the apparent lag, but nominal molecular weight alone cannot predict either the lag or the eventual concentration ratio [26,54,55,56]. Injury, altered perfusion, and the extraction method can change these parameters during a study. This conceptual model explains why a 20 min lag observed for one small-molecule microneedle sensor cannot be assigned to a 50–70 kDa neuroprotein.

## 4. Mechanisms Governing the Appearance of Neuroinjury Proteins Outside the CNS

Release, neurofluid exchange, drainage, barrier transfer, and peripheral handling act in parallel and can change with injury state. Section 4 examines these mechanisms in detail, beginning with the cellular events that generate the proteins introduced above.

### 4.1. Biomolecular Mechanisms of Traumatic Brain Injury and Biomarker Release

Traumatic brain injury initiates a temporally evolving sequence of primary mechanical and secondary biochemical events that determines which biomarkers are released, when they become detectable, and how long their signals persist [59,60]. Rapid tissue deformation, compression, stretch, and rotational shear can produce focal contusion, membrane mechanoporation, and diffuse axonal injury [59,60]. The immediate consequences include ionic disequilibrium, excessive glutamate release, intracellular calcium accumulation, mitochondrial dysfunction, energy failure, and oxidative and nitrosative stress. Calcium-dependent calpain activation, together with caspase-mediated apoptotic signaling and other proteolytic processes, promotes cytoskeletal breakdown, while necrotic and apoptotic injury releases intact and fragmented proteins into brain ISF [60].

Secondary injury develops over hours to days and can sustain biomarker production after the initiating mechanical event has ended. Damage-associated molecular patterns activate microglia and astrocytes, recruit peripheral leukocytes, and amplify cytokine and complement signaling; depending on timing and context, these responses may contribute to both tissue injury and repair [61]. Concurrent endothelial, pericyte, and tight-junction injury can disrupt the neurovascular unit, impair cerebral autoregulation, promote microthrombosis and edema, and alter BBB permeability [62]. These interacting inflammatory, metabolic, and vascular processes can therefore change both the rate of CNS biomarker release and the efficiency with which CNS-derived proteins reach CSF and blood.

The resulting biomarker profile reflects the cellular structures and molecular pathways affected. Neuronal cell-body injury produces an early release of cytoplasmic proteins such as UCH-L1 and NSE, whereas astrocytic membrane and cytoskeletal disruption, followed by reactive astrogliosis, contributes to the release of GFAP and S100B. Axonal stretch and impaired axoplasmic transport destabilize microtubules and neurofilaments, promoting tau release, amyloid precursor protein accumulation, and the delayed appearance of NfL as axonal degeneration and Wallerian processes evolve. Human high-dimensional plasma proteomics has demonstrated that acute TBI produces coordinated changes spanning neuronal, astroglial, inflammatory, metabolic, autophagy-related, tau, and amyloid-associated proteins, with several signals related to subsequent lesion burden or white-matter injury [63]. Calpain-mediated proteolysis further generates breakdown products of αII-spectrin, tau, GFAP, and UCH-L1 [64], while diffuse axonal injury can produce slower and more persistent NfL, tau, and SNTF trajectories [59,65,66,67]. Extracranial sources remain important confounders for S100B and NSE [3,68]. Thus, the concentration ultimately measured in CSF, blood, or dermal ISF represents not only injury magnitude but also the evolving cellular source, molecular processing, barrier state, compartmental transport, and clearance of each analyte.

### 4.2. ISF–CSF Exchange: The Glymphatic System and Perivascular Clearance

The glymphatic model describes one route of exchange between CSF and brain ISF. CSF enters periarterial spaces and exchanges with interstitial solutes in a process influenced by vascular pulsatility, sleep state, and AQP4 polarization; efflux is linked to perivenous, meningeal, and other drainage pathways [7,8]. Primary microdialysis studies show that brain-ISF Aβ increases with wakefulness and acute sleep deprivation [69], whereas sleep deprivation increases brain-ISF tau in mice and CSF tau in humans [70]. These state-dependent kinetics do not establish bulk glymphatic clearance as the sole mechanism. The relative roles of bulk or convective movement, dispersion, and diffusion remain under active investigation [71,72]. Accordingly, glymphatic transport should not be treated as the quantitatively dominant route for every protein. AQP4-loss experiments nevertheless show brain-wide interstitial stagnation and impaired clearance of tau and Aβ, supporting a material role for AQP4-dependent neurofluid organization [73].

Following TBI, loss of perivascular AQP4 polarization can impair solute transport and alter the residence time of injury-related molecules in brain ISF, with downstream effects on CSF and blood kinetics [11]. Related AQP4 and neurofluid abnormalities have been reported in AD and Huntington’s disease [74,75], supporting a shared but not necessarily identical clearance phenotype across acute and chronic disorders [11,76,77]. Age-related disruption of extracellular-space organization may also impair interstitial drainage independent of overt neurodegeneration [78]. Enlarged perivascular spaces are therefore of interest as imaging correlates, but they are nonspecific and are influenced by vascular and white-matter pathology [79,80,81]. Automated quantification may improve reproducibility without converting this indirect feature into a direct measure of glymphatic flux [82].

### 4.3. Meningeal Lymphatics and Cervical Lymph Drainage to the Peripheral Circulation

A portion of interstitial and CSF outflow reaches dural meningeal lymphatic vessels, which connect to deep cervical lymph nodes and ultimately the peripheral venous circulation [18,50,51]. Experimental evidence indicates that tau, amyloid-beta, alpha-synuclein, and injury biomarkers can move along glymphatic–lymphatic routes, so changes in drainage may alter the timing and magnitude of their appearance in blood [15,17]. Meningeal lymphatic function also changes with age and disease, potentially contributing to compartment-specific accumulation [83].

CSF also leaves the cranial compartment through arachnoid, perineural, and other outflow pathways whose relative contributions may vary with physiology and disease [71]. These partially overlapping routes, together with barrier-mediated transfer, should be treated as a network rather than as a fixed hierarchy for peripheral biomarker appearance [14,84].

### 4.4. Blood–Brain Barrier and Blood–CSF Barrier Transport

The blood–brain barrier (BBB) is formed by brain microvascular endothelial tight junctions and is supported by pericytes, astrocytic endfeet, and other neurovascular unit components [85,86]. The blood–CSF barrier (BCSFB) is located principally at the choroid plexus epithelium. Protein movement across these interfaces depends not only on molecular size but also on charge, conformation, binding, active transport, vesicular pathways, proteolysis, and barrier state. Smaller proteins may cross a disrupted barrier more readily, but size thresholds alone cannot predict the appearance of S100B, UCH-L1, GFAP, NfL, or tau in blood. TBI, stroke, and neuroinflammation can increase permeability and change efflux pathways [87,88]. When CSF is available, the CSF/serum albumin quotient (QAlb) helps contextualize blood–CSF barrier function and CSF flow because albumin is blood-derived [46]. Peripheral biomarker concentrations may therefore reflect a changing mixture of release, physiological drainage, barrier leak, and clearance rather than a single ‘drain’ or ‘leak’ mechanism [7,88].

### 4.5. Peripheral Clearance and Plasma Half-Lives

A peripheral signal persists for different lengths of time depending on when a protein is released, how it redistributes and is cleared, and which molecular form an assay detects. After injury, reported measurement windows tend to be short for S100B and UCH-L1, intermediate for GFAP, NSE, and some tau assays, and longer for NfL [52,89,90]. Values sometimes labelled “half-lives”—minutes to hours for S100B, approximately 7–10 h for UCH-L1, around a day for GFAP or NSE, and days to weeks for NfL—often describe a composite post-injury decline rather than a measured intrinsic systemic elimination constant. Repeated release can extend a blood trajectory even when circulating protein is removed rapidly. This distinction matters if plasma–dISF transfer parameters are to be estimated from paired samples: the disappearance curve cannot simply be treated as a fixed property of the molecule [21,22,66,91].

## 5. Biomarker-Specific Kinetic Phenotypes Across Compartments

**Figure 2:**
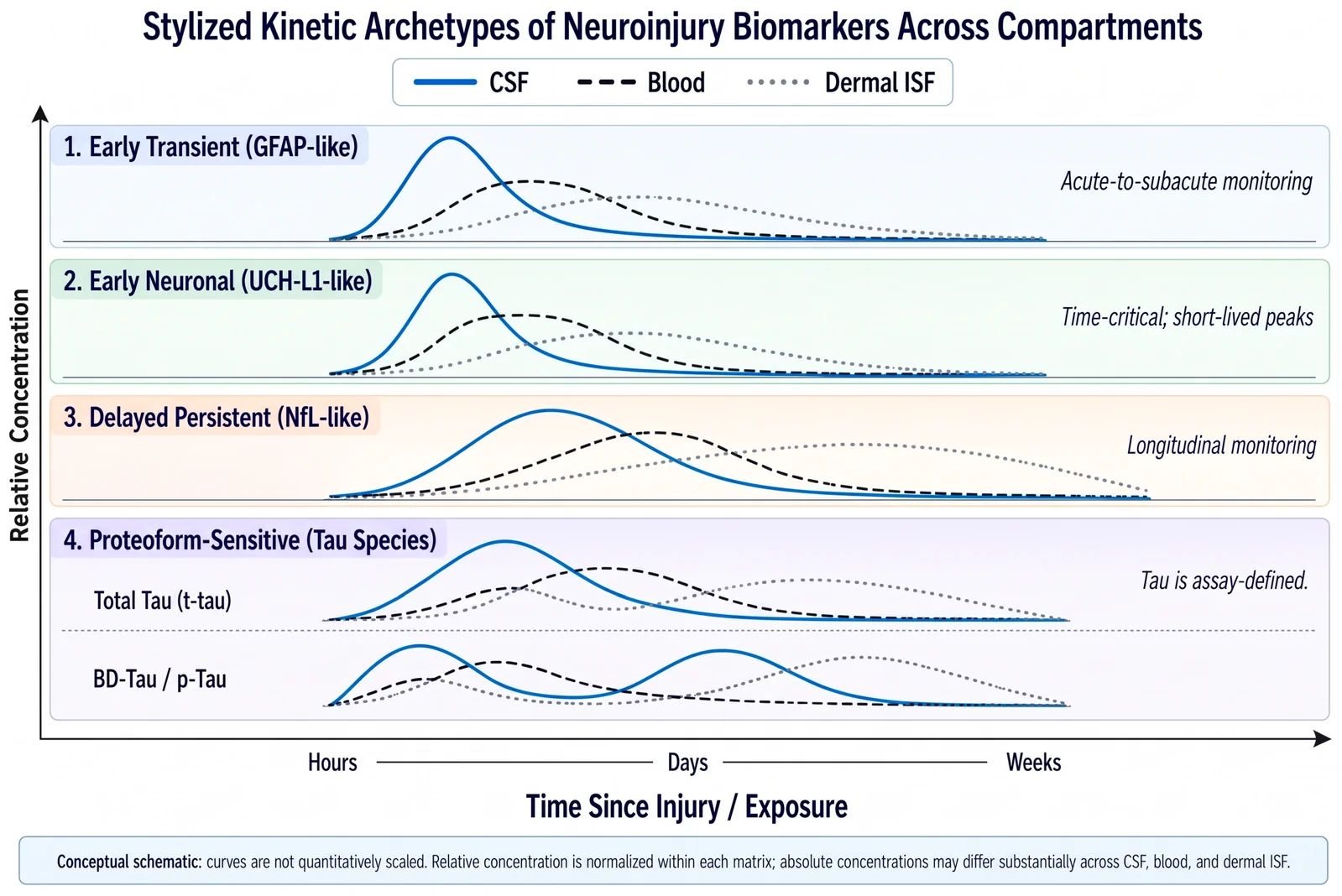
Conceptual kinetic archetypes of neuroinjury biomarkers across cerebrospinal fluid, blood, and dermal interstitial fluid. Stylized curves illustrate how the timing, amplitude, and persistence of neuroinjury biomarker signals may differ across cerebrospinal fluid (CSF; blue solid line), blood (black dashed line), and dermal interstitial fluid (dermal ISF; grey dotted line) as a consequence of cellular release, compartmental transport, barrier passage, systemic dilution, molecular processing, clearance, and sampling characteristics. An early transient astroglial pattern, illustrated using a GFAP-like profile, is characterized by a rapid proximal rise followed by a delayed and attenuated peripheral signal, with additional temporal smoothing expected in dermal ISF. An early neuronal pattern, illustrated using a UCH-L1-like profile, emphasizes the narrow sampling window associated with short-lived peripheral elevations and the risk that time-integrating microneedle systems may attenuate brief peaks. A delayed and persistent axonal pattern, illustrated using an NfL-like profile, is more compatible with longitudinal and integrator-type dermal ISF sampling because concentrations remain elevated over days to weeks. Tau-family profiles are shown separately to emphasize that total tau, brain-derived tau, and phosphorylated tau species represent distinct assay-defined analytes whose cross-compartment kinetics may differ because of secretion, fragmentation, post-translational modification, epitope selection, transport, and clearance. Curves are conceptual illustrations informed by the reported differences in blood-biomarker time courses [52,66,89,90], not curves fitted to a specific paired-compartment dataset. Each curve is normalized for display. The “Relative Concentration” axis therefore conceals potentially very large absolute concentration differences among CSF, blood, and dISF; equal plotted heights do not indicate equal concentrations. No fixed peak times, measured dISF kinetics, or direct quantitative relationships between compartments are implied. Abbreviations: BD-tau, brain-derived tau; CSF, cerebrospinal fluid; GFAP, glial fibrillary acidic protein; ISF, interstitial fluid; NfL, neurofilament light chain; p-tau, phosphorylated tau; t-tau, total tau; UCH-L1, ubiquitin C-terminal hydrolase-L1.

Neuroinjury biomarkers have distinct kinetic phenotypes across brain ISF, CSF, blood, and dISF because they differ in cellular source, molecular form, stability, binding, transport, and clearance. These features determine both detectability and the sampling cadence required for dISF studies. GFAP and NfL are rational first candidates for paired validation because of their CNS enrichment and sustained blood signals; UCH-L1 provides a demanding test of hyperacute sampling. S100B, NSE, and inflammatory proteins are valuable contextual markers but have substantial extracranial or systemic confounding. These priorities are hypotheses for validation, not evidence that established neuroinjury proteins are already clinically measurable in dISF. Figure 2 illustrates kinetic archetypes, and Table 1 summarizes compartment-aware expectations.

### 5.1. GFAP: Astrocytic Injury Signature

GFAP (49–51 kDa) is a type III intermediate filament expressed predominantly in astrocytes and is among the most CNS-enriched neuroinjury proteins in clinical use [105,106,107]. In TBI, plasma GFAP rises within hours, commonly peaks during the first day, and can remain elevated for several days depending on injury severity and assay [52,108]. Clinical implementation of combined blood GFAP/UCH-L1 testing marked an important translational milestone [108,109]. Beyond neurotrauma, plasma GFAP is associated with amyloid pathology and clinical progression in AD [110]. These features make GFAP a strong candidate for the first paired plasma–dISF studies, but they do not establish its current clinical detectability or concordance in microneedle-collected dISF [111].

### 5.2. UCH-L1: Hyperacute Neuronal Signal

UCH-L1 (25 kDa) is a neuronal cytoplasmic protein that accounts for 1–2% of total soluble brain protein and is among the earliest detectable peripheral biomarkers of brain injury [52,112]. Following TBI, plasma UCH-L1 becomes detectable within 1 h of injury and typically declines over the ensuing hours, although an approximately 7–10 h observed decline should not be equated with an independently measured systemic elimination half-life. In mild TBI, UCH-L1 is undetectable beyond 24–48 h, making it particularly useful as a marker of acute injury [21,22,52]. These transient kinetics may limit compatibility with integrator-type dermal ISF microneedle sampling approaches and instead require short-cadence sampling strategies.

### 5.3. NfL: Sustained Axonal Degeneration Marker

Neurofilament light chain (NfL, 68 kDa) is an axonal cytoskeletal protein released following axonal injury and thought to reach peripheral blood through glymphatic and lymphatic pathways [113]. Its prolonged post-injury blood trajectory, often lasting weeks, makes it useful for monitoring subacute and cumulative axonal injury; this duration should not be interpreted as a measured intrinsic systemic half-life [66,89]. Following TBI, plasma NfL rises progressively over 1–2 weeks and remains elevated for months in proportion to total axonal burden [66,67]. NfL is among the most extensively validated plasma biomarkers across neurodegenerative and neuroinflammatory diseases, including multiple sclerosis, ALS, frontotemporal dementia, and preclinical AD [114,115]. Relative to more transient biomarkers, NfL’s slow, persistent kinetics are particularly compatible with integrator-type dermal ISF microneedle sampling approaches [25,116,117].

### 5.4. Tau Species: Epitope- and Proteoform-Dependent Kinetics

Tau biomarkers comprise multiple assay-defined proteoforms and fragments whose release, secretion, transport, and clearance are not interchangeable [19,20]. Human brain microdialysis has detected total tau and p-tau181 and demonstrated early extracellular tau dynamics after severe TBI and in neurosurgical patients [41,42,43]. Sleep deprivation also increases brain-ISF tau in mice and CSF tau in humans [70], reinforcing the need to account for physiological state. In TBI, impaired neurofluid transport may contribute to tau retention and propagation [11,76]. In AD, plasma p-tau217 and p-tau181 show strong diagnostic performance for amyloid-related pathology [93,94]. Microneedle platforms targeting p-tau181, p-tau217, or Alzheimer-related nucleic acid signals demonstrate analytical or preclinical feasibility [95,96,118]; none yet establishes clinical dISF–blood or dISF–CSF concordance for tau proteoforms.

### 5.5. S100B and NSE: Utility and Extracranial Confounds

S100B (10.5 kDa) and neuron-specific enolase (NSE, 78 kDa) were among the earliest clinically adopted neuroinjury biomarkers but are limited by substantial extracranial expression. S100B is also expressed in adipose tissue, skeletal muscle, melanocytes, and Schwann cells [68], whereas NSE is abundant in erythrocytes and neuroendocrine cells [98]. Although NSE remains incorporated into post-cardiac-arrest frameworks, both biomarkers are vulnerable to confounding from polytrauma, hemolysis, and peripheral tissue injury [97,99]. A microneedle immunoassay detected S100B in dISF from melanoma-expressing mice [101], demonstrating analytical accessibility but also illustrating why a skin measurement cannot be assumed to be CNS-derived. For dISF applications, background tissue expression substantially limits CNS specificity.

### 5.6. Amyloid-β (Aβ40, Aβ42) and Aβ42/40: Clearance- and Compartment-Sensitive Example

Amyloid-β (Aβ) peptides are cleavage products of amyloid precursor protein whose concentrations are influenced by production, aggregation, transport, and clearance. In vivo microdialysis established soluble brain-ISF Aβ metabolism and half-life [39], and human studies showed that brain-ISF Aβ dynamics vary with neurological status after acute injury [40,41]. Aβ is also regulated by the sleep–wake cycle [69]. The Aβ42/Aβ40 ratio is generally more informative than either absolute concentration because it reduces some preanalytical and interindividual variability [102]. In dISF, Aβ should presently be regarded as an exploratory transport- and assay-validation target rather than a quantitative measure of CNS injury; sensitivity, proteoform identity, and paired-compartment relationships remain to be established [23].

### 5.7. Cytokines and Inflammatory Proteins: High Relevance, Lower CNS Specificity

Inflammatory proteins, including interleukins, chemokines, and acute-phase reactants, are frequently measured alongside structural neuroinjury biomarkers and provide important contextual information regarding neuroinflammatory and systemic immune states. In brain ISF and CSF, inflammatory protein dynamics may reflect evolving neuroinflammatory responses following injury [28,32,33]. In blood and dermal ISF, however, these proteins are strongly influenced by systemic immune responses, peripheral tissue injury, infection, and physiological variation, substantially limiting CNS specificity. Acute exercise is a particularly well-quantified example of this physiological variation: a recent meta-analysis of 1826 participants found that IL-6 rises substantially after acute exercise (pooled response ratio 2.15, 95% CI 1.50–3.07), alongside smaller but significant increases in creatine kinase and lactate dehydrogenase, underscoring that exercise alone can produce inflammatory and muscle-injury signal changes that could be mistaken for neuroinflammatory or systemic injury responses [119]. Inflammatory proteins, together with clinical symptoms and individualized contextual data, can help interpret CNS biomarker findings within broader systemic and inflammatory contexts. Dedicated microneedle electrochemical immunosensors for such inflammatory markers have already reached sub-nanogram sensitivity outside the CNS-injury context, including aptamer-based C-reactive protein sensors with detection limits near 1 ng/mL and interleukin-6 immunosensors reaching 0.54 pg/mL, illustrating an analytical floor that dermal ISF neuroinflammatory panels would need to match [116].

### 5.8. mTBI, Sport-Related Concussion, and Exercise-Related Biomarker Variation

Mild traumatic brain injury (mTBI) and sport-related concussion are biologically heterogeneous, and blood biomarker interpretation is strongest when structural, inflammatory, clinical, and imaging measures are integrated rather than treated as interchangeable single-analyte tests [120]. In collegiate athletes, peroxiredoxin-6 (PRDX-6) was elevated during the subacute phase of sport-related concussion, whereas PRDX-6 and total tau contributed to separation from controls at medical clearance, suggesting that physiological restitution may extend beyond symptom-based recovery [121]. A separate multimodal study linked post-concussion variation in total tau, PRDX-6, monocyte chemoattractant protein-4 (MCP-4), cerebral blood flow, and resting-state connectivity, supporting biomarker–imaging co-interpretation rather than isolated thresholding [122]. Systemic inflammatory profiles also showed concussion-associated elevations in MCP-4 and macrophage inflammatory protein-1β (MIP-1β) that differed from musculoskeletal-injury patterns, while monocyte chemoattractant protein-1 (MCP-1) and MCP-4 were associated with days to recovery [123]. By contrast, plasma IL-6 did not differ independently between concussed and healthy athletes and was not associated with symptom burden or recovery time; an interaction with prior concussion history indicates that exposure history can modify an otherwise weak group-level signal [124].

Baseline and exercise effects further complicate longitudinal monitoring. In healthy athletes, multiple previous concussions were associated with sex-specific MCP-1 or MCP-4 patterns and collision-sport participation with higher tau in males, although extracranial effects of exercise and muscle injury could not be excluded [125]. In healthy men, a single high-intensity interval-training bout increased 10 of 12 candidate markers measured, including S100B, NSE, BDNF (Brain-Derived Neurotrophic Factor), neurogranin, PRDX-6, CK-BB (Creatine Kinase-BB), von Willebrand factor, MCP-1, MMP-9 (Matrix Metalloproteinase-9), and total tau; after six sessions, several acute responses were attenuated, and total tau no longer increased significantly [126]. Together with the broader acute-exercise meta-analysis [119], these findings support standardized pre-sampling activity windows, training-load documentation, and within-person baselines in both blood and dermal-ISF studies. Exercise-associated changes should be treated as physiological modifiers and possible extracranial confounds, not as evidence of occult CNS injury.

## 6. State-of-the-Art Measurement Technologies

Advances in neuroinjury biomarker biology and analytical technology have substantially expanded the ability to measure low-abundance CNS-derived proteins in blood and other low-volume biological matrices. Translation of these approaches to dermal interstitial fluid (dISF) depends not only on analytical sensitivity but also on molecular specificity, sample-volume requirements, recovery, matrix effects, dynamic range, and compatibility with microneedle collection and extraction workflows. These considerations are particularly important because recoverable dISF volumes may be in the sub-microlitre to low-microlitre range, protein concentrations may differ markedly from those in plasma, and microneedle devices may introduce selective extraction, adsorption, dilution, or enrichment effects. Accordingly, no single analytical platform is likely to be optimal across discovery, verification, and targeted biomarker quantification. Complementary technologies—including ultrasensitive digital immunoassays, high-plex affinity proteomics, emerging ultrasensitive multiplex platforms, mass spectrometry, and integrated biosensors—can serve different roles in characterizing dISF and validating candidate neurological biomarkers for minimally invasive monitoring.

### 6.1. Ultrasensitive Digital Immunoassays (Simoa)

Single-molecule array (Simoa; Quanterix) is an ultrasensitive digital immunoassay technology that isolates and detects individual immunocomplexes in femtolitre-scale reaction wells, providing sensitivity up to approximately three orders of magnitude greater than conventional automated immunoassays [127,128]. The current Simoa HD-X platform supports both single-analyte and multiplex assays and has an extensive neurology-focused assay portfolio encompassing established and emerging CNS biomarkers, including GFAP, neurofilament light (NfL), UCH-L1, total tau, brain-derived tau (BD-tau), amyloid-β40 and amyloid-β42, and phosphorylated tau species including p-tau181, p-tau205, and p-tau217. Contemporary multiplex configurations include the Neurology 4-Plex D assay, which simultaneously quantifies BD-tau, NfL, GFAP, and UCH-L1, and the Neurology 4-Plex E assay for Aβ40, Aβ42, GFAP, and NfL [129]. These capabilities support targeted validation and longitudinal quantification of low-abundance neurological biomarkers identified through broader discovery-proteomic approaches.

The principal strength of Simoa for dISF applications is its combination of very high analytical sensitivity with quantitative, antibody-based measurement of predefined biomarkers. However, its suitability for microneedle-derived dISF cannot be inferred directly from performance in plasma, serum, or CSF. Matrix-specific validation should establish parallelism, dilutional linearity, spike recovery, precision, interference, analyte stability, adsorption losses, and matrix-specific limits of detection and quantification. Sample-volume requirements also need to be considered carefully because nominal assay input volumes may exceed the native volume obtainable from individual microneedle collections, necessitating elution, dilution, pooling, or optimized low-volume workflows. These manipulations may in turn influence analytical sensitivity and apparent biomarker concentration.

Simoa may therefore be useful for targeted validation after candidate biomarkers have been identified by high-plex proteomic methods, particularly when longitudinal quantification of selected CNS-derived proteins is required. Establishing concordance among dISF, plasma, CSF, and, where available, brain ISF measurements will be essential before Simoa-derived dISF concentrations can be interpreted as quantitative surrogates of CNS injury or pathology.

### 6.2. High-Plex Discovery Proteomics: PEA, Aptamer-Based, and Emerging Platforms

High-plex proteomic technologies now enable discovery-scale measurement of several thousand proteins from very small biofluid volumes and provide an increasingly powerful approach for characterizing the molecular composition of interstitial fluid (ISF). Proximity extension assay (PEA) coupled with next-generation sequencing, implemented in the current Olink Explore HT platform, has expanded beyond the earlier 3072-protein configuration and now assays more than 5400 proteins across a broad range of biological pathways using microlitre-scale sample volumes [104,130,131]. Complementary affinity-based technologies provide even greater proteome coverage; for example, the SomaScan 11K aptamer-based platform generates approximately 11,000 protein measurements representing approximately 10,000 unique human proteins [132], while emerging affinity- and mass-spectrometry-based approaches continue to increase proteomic depth. PEA has already been applied to cerebral microdialysate in severe TBI, demonstrating that longitudinal neuroinflammatory, vascular, metabolic, and tissue-injury protein dynamics can be characterized directly within brain ISF [28,133], and large-scale PEA profiling has also been successfully performed in CSF. Extending such discovery proteomics across matched brain ISF, CSF, blood, and dermal ISF samples could provide a powerful means of defining compartment-specific signatures and determining how neurological proteins distribute, appear, and change across biological compartments. These approaches are therefore attractive for characterization of microneedle-collected dermal ISF, but require rigorous matrix-specific analytical validation because platforms optimized primarily for plasma or serum may show different recovery, detectability, interference, and dynamic-range characteristics in low-protein or non-standard matrices [134]. Additional considerations include sample-volume requirements, limits of detection and quantification, missingness at low abundance, batch and plate effects, normalization procedures, assay- or panel-version bridging, and platform-specific measures of relative abundance (e.g., Olink NPX or SomaScan relative fluorescence units) rather than absolute protein concentrations. Cross-platform differences in affinity reagents, epitope accessibility, proteoform recognition, and analytical sensitivity should also be considered when comparing results between technologies or biological compartments [135].

### 6.3. Mass Spectrometry for Proteoforms and Fragments

Mass spectrometry characterizes proteins and peptide fragments based on mass-to-charge properties, enabling detailed analysis of proteoforms and fragment characterization. This is particularly important for tau-family analytes, where immunoassay epitope coverage varies substantially across platforms. Immunoprecipitation–mass spectrometry has achieved the sensitivity required for plasma Aβ42 measurement and validated the plasma Aβ42/Aβ40 ratio as an AD biomarker [103]. Extracellular vesicle proteomic profiling of tau isoforms and brain-specific peptide sequences in CSF and blood is advancing the field beyond antibody–epitope limitations [136]. Table 2 compares the practical sampling and assay constraints across the four principal compartments and suction-blister ISF.

## 7. Microneedle Dermal ISF Sampling: Devices, Workflows, and Validation

**Figure 3:**
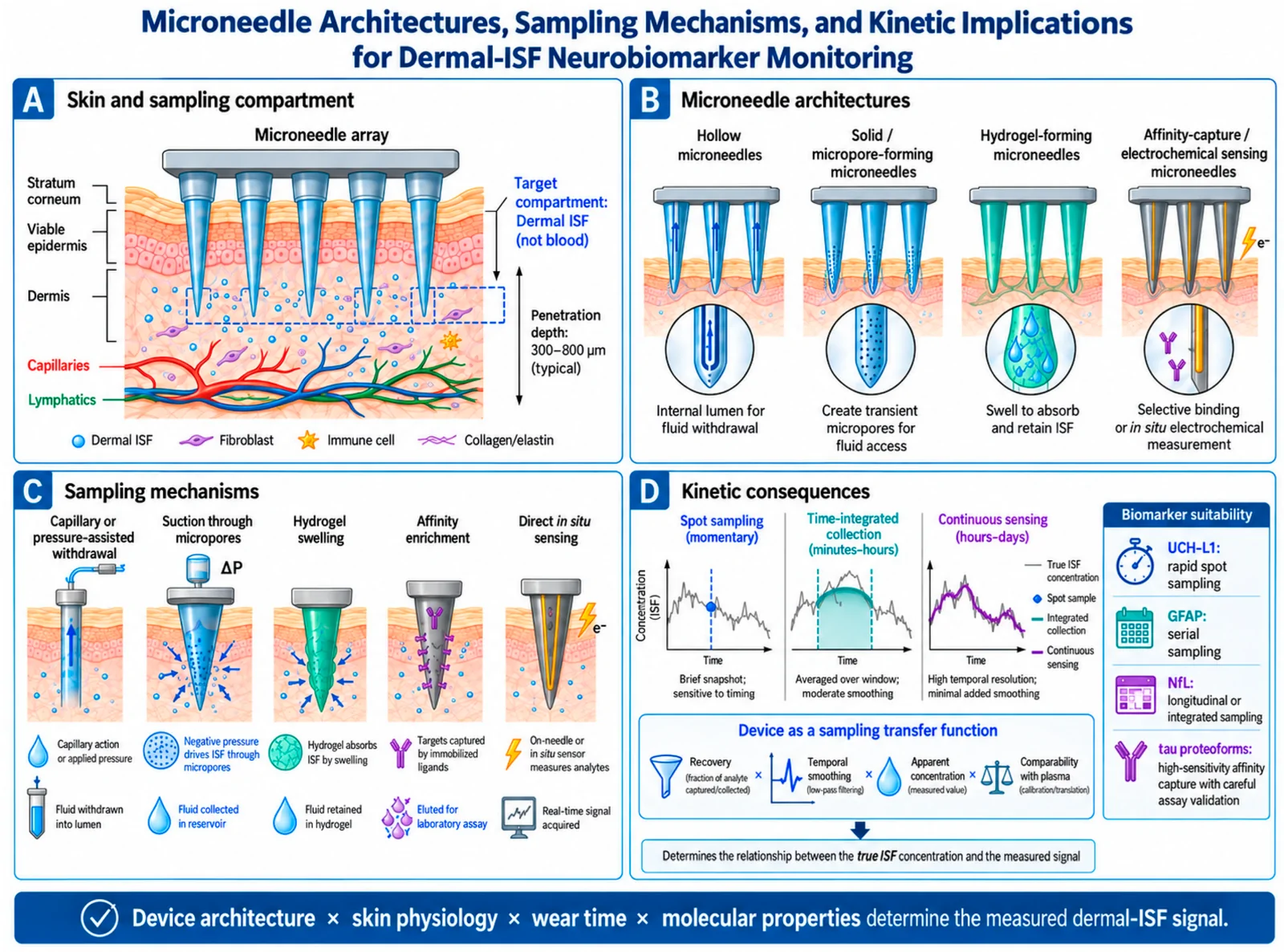
Microneedle architectures, sampling mechanisms, and kinetic implications for dermal interstitial fluid biomarker monitoring: (**A**) Schematic of the skin sampling compartment, showing the stratum corneum, viable epidermis, dermis, dermal interstitial fluid (dISF), capillaries, and lymphatics. Microneedles are designed to traverse the stratum corneum and access superficial dermal ISF while minimizing direct vascular sampling. (**B**) Representative microneedle architectures include hollow microneedles for direct fluid withdrawal; solid or micropore-forming microneedles that create transient pathways for subsequent ISF extraction; hydrogel-forming microneedles that swell and retain ISF over a defined wear interval; and affinity-capture or electrochemical sensing microneedles that enable selective analyte enrichment or direct in situ measurement. (**C**) Principal sampling mechanisms include capillary- or pressure-assisted withdrawal, suction through micropores, hydrogel swelling, affinity enrichment, and direct in situ sensing. These approaches differ in collected volume, analyte recovery, temporal integration, and downstream analytical workflow. (**D**) Conceptual kinetic consequences of device format, contrasting momentary spot sampling, time-integrated collection, and continuous sensing. Illustrative matching of device kinetics to neurobiomarker behaviour includes rapid or high-frequency sampling for UCH-L1, serial sampling for GFAP, longitudinal or integrator approaches for NfL, and high-sensitivity affinity-based approaches for selected tau proteoforms, subject to analyte-specific analytical validation. Because temporal offset and smoothing depend on the analyte, device, perfusion, wear interval, and physiological state, a fixed plasma–dISF relationship should not be assumed. The microneedle platform therefore acts as a sampling transfer function, with device architecture, skin physiology, wear time, and molecular properties influencing analyte recovery, temporal smoothing, apparent concentration, and comparability with plasma.

Microneedle (MN)-enabled dISF sampling provides a minimally invasive route for repeated biomarker collection from skin interstitium. Solid, hollow, porous, and hydrogel-forming architectures differ in extraction mechanism, recovery, temporal integration, and apparent concentration [23,116,117,146]. They traverse the stratum corneum and enter superficial skin with substantially less bleeding and discomfort than conventional venipuncture, although tolerability and skin effects remain device- and use-dependent. Figure 3 compares the skin compartment, common microneedle architectures, extraction mechanisms, and the resulting sampling intervals.

### 7.1. Microneedle Types and Characteristics

Hollow microneedles enable collection of relatively large volumes (0.1–10 µL) suitable for multi-analyte analysis, although they are prone to clogging [147,148,149]. Human hollow-MN extraction has supported proteomic and transcriptomic analysis and characterization of dISF extracellular vesicles [140]. Solid microneedles create micro-channels through which ISF is absorbed by an overlying matrix, typically yielding 0.1–1 µL, requiring elution for highly sensitive platforms but often providing insufficient native volume for conventional ELISA [23,54]. Multiple engineering strategies have been explored to increase extraction efficiency, including vacuum-assisted systems and laser pre-treatment of skin [139,150,151]. In humans, a vacuum-assisted MN patch recovered an average of 20.8 µL in 25 min and identified more than 600 medically relevant proteins [141], while a volume-metered single-MN device supported multianalyte measurement and dried-paper sample storage [142].

Hydrogel-forming microneedle arrays use swellable polymers to absorb dISF after insertion; mesh density and swelling determine which proteins enter and how readily they can be eluted [117]. Swellable patches may be suitable for repeated sampling and integrate signal over a defined wear interval [152]. Porous polymer needles with anti-adhesive surfaces and anisotropic silk needles offer different routes to rapid fluid uptake [153,154]. The silk results come from mouse skin; they do not establish recovery of CNS-derived proteins from human dISF. Prototype hydrogel systems have supported extended monitoring in artificial ISF and bioelectronic recording in vivo [155,156], while broader reviews emphasize insertion mechanics, manufacturing, sterilization, and regulatory constraints [157]. Recent electrochemical, materials, extraction, hydrogel, and physiological-fluid reviews converge on the need for device-specific calibration, antifouling strategies, mechanical and wear validation, scalable manufacturing, and human clinical benchmarking rather than extrapolation from artificial matrices or animal models [158,159,160,161,162]. Sampling mechanisms include capillary withdrawal through hollow needles, pressure-gradient extraction, hydrogel swelling, fluid passage through porous needles, electro-assisted enrichment, and affinity capture. Hollow and porous channels may clog or retain protein; hydrogel networks can exclude or bind larger analytes; and surface capture can enrich a target while making total extracted volume difficult to define [57]. As an equipment-light example, a one-press hollow-MN paper patch integrated passive collection and colorimetric measurement of glucose, uric acid, lactate, and pH within seven minutes [163]. That study used biomimetic and ex vivo porcine-skin models; it demonstrates engineering feasibility, not human clinical or neuroprotein validation.

### 7.2. ISF Volume, Protein Detection, and Analytical Validation

A low-abundance neurological protein may be lost before it reaches the assay. Proteins at pg/mL concentrations can adsorb nonspecifically to needle polymers, hydrogel networks, collection surfaces, and extraction tubes; losses can be large relative to the collected mass [29,57,117,153]. Researchers should measure blank-device background, recovery across clinically relevant concentrations, adsorption and elution efficiency, dilutional parallelism, matrix interference, and stability for each material–analyte–assay combination. Low-binding surfaces, validated surface coatings, and assay-compatible surfactants may reduce adsorption, but each treatment must be checked for interference with affinity binding or electrochemical detection. Affinity capture and metal–hydrogel platforms demonstrate encouraging analytical sensitivity [164,165,166]; sensitivity in a spiked matrix does not establish quantitative recovery of CNS-derived proteins from human skin.

An assay result from a microneedle eluate is not automatically the absolute concentration in native dISF. The measured amount depends on the fraction that moved from tissue into the device, the volume or duration sampled, elution efficiency, any dilution, and the final assay response. In cerebral microdialysis, relative recovery is defined against an independently estimated interstitial concentration; zero-net-flux approaches can sometimes estimate that concentration by varying perfusate levels [29]. The principle is useful for microneedle validation, but zero-net-flux calibration cannot simply be transplanted to an absorbing patch. For each device, investigators should report recovered mass and volume or another justified normalization denominator, perform matrix-matched calibrations and spike-recovery experiments, and test whether endogenous reference proteins remain sufficiently stable to correct for collection variation. An endogenous normalizer can reduce device-to-device variability only after its own partitioning and recovery are validated; it cannot establish an absolute interstitial concentration by assumption [23,26,117].

Human dISF contains a broad molecular repertoire, but overlap with plasma does not imply fixed quantitative equivalence. Microneedle-assisted proteomics identified many shared proteins [54], and hollow-MN, vacuum-assisted, and volume-metered devices have extended human protein profiling and practical sample handling [140,141,142]. A 21-participant study using microneedle-created micropores plus mild suction found clinically relevant metabolites and other molecules in dISF, with both shared and matrix-distinct features relative to paired plasma [167]. In 20 volunteers, selected systemic inflammatory and cardiovascular proteins showed variable dISF–serum relationships [111], and protein measurement has also been demonstrated in heart failure and melanoma applications [138,168]. A recent cardiovascular-focused review similarly concluded that real-time dISF protein monitoring remains promising but requires paired blood–dISF validation, sensitive assays, and control of local physiology before clinical interpretation [169]. Paired blister-ISF and plasma cytokines in vitiligo showed both shared systemic signals and strong local tissue effects [143]. Paired forearm skin dialysate and plasma metabolomics found 39 correlated metabolites alongside 12 metabolites unique to the skin dialysate [144]. In rats, MN-collected dISF reproduced vancomycin pharmacokinetic profiles and supported measurement of polio-specific antibodies [170]. Most recently, paired dISF and plasma from five healthy volunteers had closely overlapping N-glycan profiles despite approximately threefold lower total protein in dISF [27]. Collectively, these studies support systemic molecular and glycomic feasibility while demonstrating analyte- and tissue-specific partitioning; they do not establish quantitative recovery of CNS-enriched injury proteins.

### 7.3. Emerging Evidence and Remaining Clinical Validation Gap

Direct clinical evidence for CNS-enriched protein biomarkers in human dISF remains limited. Human studies establish tolerable collection, broad molecular coverage, and analyte-specific overlap with paired blood [140,141,142,167], but none validates GFAP, UCH-L1, NfL, tau, or Aβ as a quantitative clinical dISF marker. Paired cytokine and metabolomic studies further show that local skin biology can create matrix-distinct signals [143,144]. More broadly, blood-to-dISF relationships for proteins are incompletely characterized, and dISF should not be assumed to be a direct quantitative proxy for blood [171]. Current neurodegeneration-oriented devices are better classified as analytical or preclinical proofs of concept: an integrated sensor measured p-tau181 and p-tau217 [96], a gold-nanoparticle MN platform targeted p-tau181 [95], a fluorescent hydrogel patch measured Alzheimer-related MALAT1 (Metastasis-Associated Lung Adenocarcinoma Transcript 1) in mice [118], and a melanoma model demonstrated dISF S100B sensing with an explicitly extracranial source [101]. These reports support technical feasibility for selected analytes and recognition chemistries, but none supplies the paired human dISF–plasma–CSF evidence needed for clinical interpretation of established neuroinjury proteins.

### 7.4. Sampling Physics, Kinetic Implications, and Device Design

Device behaviour should be matched to biomarker kinetics. Hollow MNs can approximate spot sampling, whereas hydrogel and affinity-capture systems integrate exposure over their wear interval [116,117]. Hyperacute UCH-L1 trajectories may be attenuated by long integration, while persistent NfL trajectories may be more compatible with serial or integrator designs. A fixed plasma–dISF lag should not be assumed: temporal offset and smoothing are analyte-, device-, perfusion-, and state-dependent [26,54,55]. Human continuous-MN precedents remain concentrated in small molecules: an integrated wearable array monitored glucose, lactate, and alcohol [172], and hollow-MN electrochemical aptamer sensors measured phenylalanine with an approximately 20 min lag after an oral bolus [173]. In a 2026 pilot clinical trial involving six healthy participants, solid-needle electrochemical aptamer patches measured vancomycin in dISF at 5 min resolution for up to 24 h; performance was strongest during the first 12 h because of sensor degradation, and compartmental modelling was required to relate dISF trajectories to plasma pharmacokinetics [174]. This is an important human feasibility milestone, but it validates continuous therapeutic-drug monitoring rather than continuous neuroprotein measurement. Continuous animal studies confirm individualized blood–ISF kinetics [175], and emerging active-reset, molecular-pendulum, resilient bioelectrode, and pressure-gradient systems illustrate possible architectures for longitudinal sensing [176,177,178,179]. These studies establish device-level precedents, not continuous human neuroprotein validation. Wear time and anatomical site should be specified together. A forearm patch and a back patch may sample skin with different stratum corneum properties, interstitial volume, vascular perfusion, and movement, and a longer wear period can both increase recovered mass and smooth a short-lived peak [23,26,57]. Repeated insertion may also cause local mechanical stress or low-grade inflammation. S100B has cutaneous sources, and IL-6 can be produced locally; an apparent baseline increase after insertion therefore needs a sham-site, adjacent-site, or time-matched control before it is attributed to the CNS [68,101,143].

### 7.5. Compartment Linkage: Paired Sampling, Three Testable Models, and Barrier Context

A central requirement for dISF translation is paired longitudinal sampling with plasma and, where clinically feasible, CSF. Such data can distinguish three testable relationships rather than presuppose one. In a rapid-exchange model, dISF tracks plasma within an analyte- and state-specific offset. In an integrator model, the device and tissue compartment smooth transient peaks over the wear interval. In a local-confound model, skin perfusion, inflammation, metabolism, or local protein expression dominates the measured signal [23,26,101,143,144,180]. Different analytes may follow different models in the same person. Study designs should therefore specify time-from-injury, collection cadence, device transfer function, skin site, and preanalytical controls rather than importing assumptions from glucose sensing.

When CSF sampling is available concurrently, interpreting the CSF–blood and CSF–dermal ISF relationships can be strengthened by barrier and transport indices such as the CSF/serum albumin quotient (QAlb), which reflects blood–CSF barrier permeability and CSF flow dynamics [46,181,182,183]. Emerging bi-component MRI modelling approaches for quantifying CSF outflow provide a complementary, non-invasive route to characterizing these flow dynamics when lumbar or ventricular sampling is not feasible [184]. Where CSF is unavailable, blood-based vascular and endothelial markers measured by multiplex proteomics (PEA/NGS) can serve as partial proxies for neurovascular state and help explain cross-compartment discordance in peripheral monitoring studies.

### 7.6. Minimum Evidence Package for MN Dermal ISF Validation

To establish MN dermal ISF as a credible matrix for neuroproteomic biomarker monitoring, the following minimum evidence package is proposed: (1) paired plasma–dermal ISF time-course studies spanning same-day kinetics and multi-day trajectories in the intended clinical population; (2) analytical validation for each target analyte and platform, including spike-recovery, dilution linearity, parallelism, and interference testing in MN eluate matrices [23,117,127]; (3) stability studies addressing wear-time dependence, elution and storage conditions, and freeze–thaw cycles; (4) test–retest reliability and baseline characterization, including within-subject variability under stable conditions to calibrate trajectory-based interpretation; (5) confound characterization studies for exercise, hydration, temperature, circadian/sleep phase, and local skin inflammation as modifiers rather than exclusion criteria; (6) device and workflow reporting standards encompassing device type and material, anatomical site, wear time, insertion and skin-response assessment, adsorption controls, elution protocol, and recovered-volume or justified normalization approach [26]; and (7) use-case alignment to biomarker kinetic phenotype, prioritizing NfL and GFAP for persistent/subacute trajectories (first-tier), carefully selected tau species where quantifiable (second-tier), and UCH-L1 as an acute feasibility test case with optimized cadence. Markers with extracranial confounders (NSE, S100B) are secondary candidates included primarily to benchmark specificity performance.

**Figure 4:**
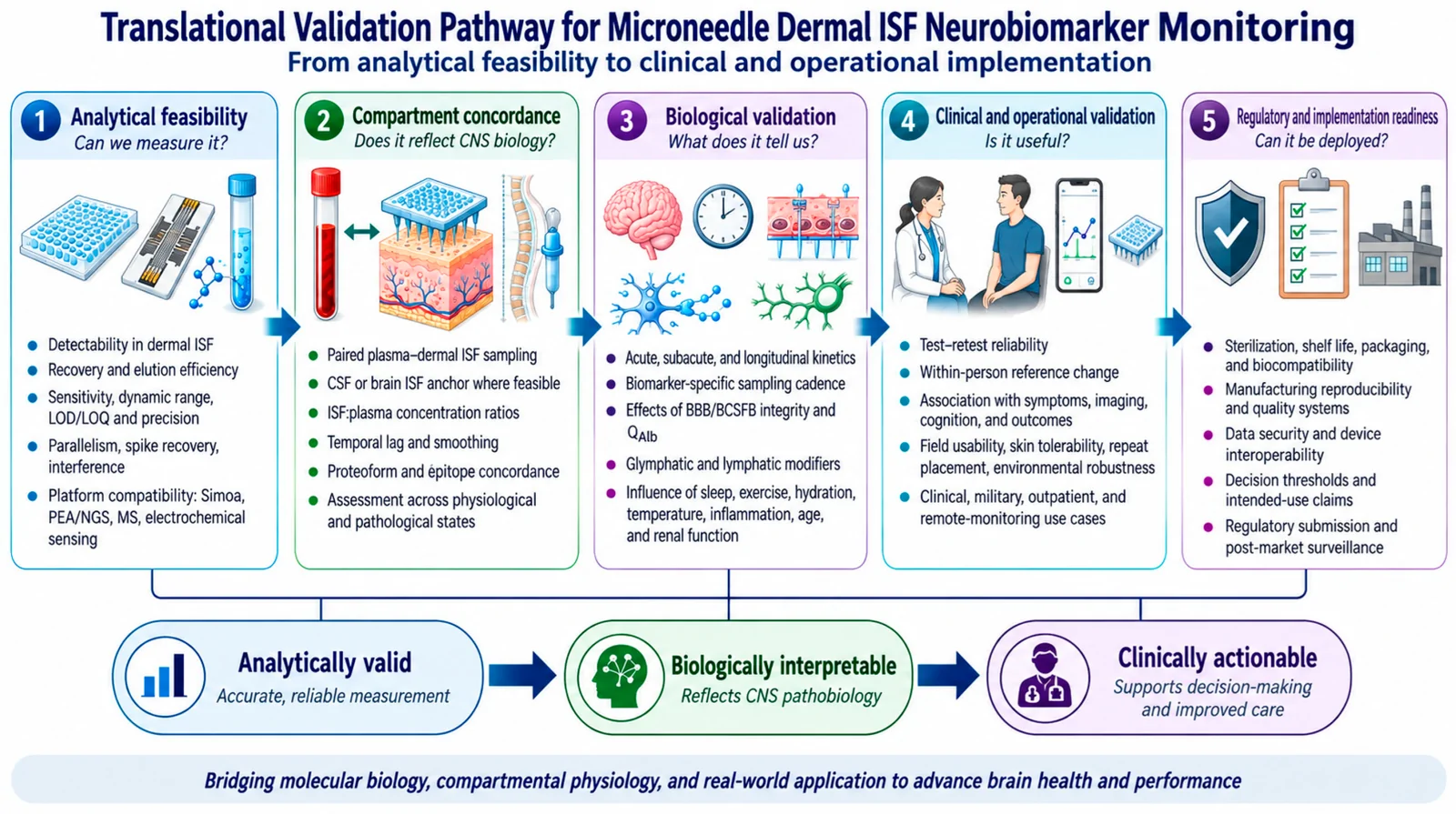
Translational validation pathway for microneedle-enabled dermal interstitial fluid neurobiomarker monitoring. A proposed framework outlining the sequential evidence required to translate microneedle dermal interstitial fluid (ISF) sampling from analytical proof-of-concept to clinically or operationally actionable neurobiomarker monitoring. The pathway is organized into five domains: (1) analytical feasibility, including detectability in dermal ISF, recovery and elution efficiency, assay sensitivity and precision, interference testing, and platform compatibility; (2) compartment concordance, including paired plasma–dermal ISF sampling, comparison with CSF or brain ISF where feasible, concentration ratios, temporal lag, smoothing effects, and proteoform or epitope concordance; (3) biological validation, including acute, subacute, and longitudinal kinetics, biomarker-specific sampling cadence, and the influence of BBB/BCSFB integrity, QAlb, glymphatic/lymphatic transport, and physiological modifiers such as sleep, exercise, hydration, temperature, inflammation, age, and renal function; (4) clinical and operational validation, including test–retest reliability, within-person reference change, associations with symptoms, imaging, cognition, and outcomes, as well as usability and tolerability under real-world conditions; and (5) regulatory and implementation readiness, including sterilization, shelf life, packaging, biocompatibility, manufacturing reproducibility, device interoperability, intended-use claims, and post-market surveillance. Together, these domains support progression from analytically valid to biologically interpretable to clinically actionable dermal ISF neurobiomarker monitoring. Abbreviations: BBB, blood–brain barrier; BCSFB, blood–cerebrospinal fluid barrier; CSF, cerebrospinal fluid; ISF, interstitial fluid; MS, mass spectrometry; PEA, proximity extension assay; QAlb, CSF/serum albumin quotient.

To help organize the evidence required for clinical and operational translation of microneedle dermal ISF neurobiomarker monitoring, Figure 4 summarizes a proposed validation pathway spanning analytical feasibility, compartment concordance, biological validation, clinical and operational performance, and regulatory readiness.

### 7.7. Safety, Biocompatibility, and Regulatory Readiness

Transdermal MN devices have shown favourable safety and tolerability across several applications, but safety evidence remains specific to material, geometry, wear time, reuse, and intended population [23,146]. Translation of a neuroprotein device will require biocompatibility, sterility, packaging and shelf-life validation, manufacturing reproducibility, analytical performance, clinical performance, human factors, cybersecurity where applicable, and an intended-use claim proportionate to the evidence. Continuous glucose monitoring provides a useful but limited precedent: the FDA cleared the first over-the-counter CGM in 2024 [185], and ISO/IEEE 11073-10425:2024 defines CGM communication and interoperability requirements [186]. CGMs measure an abundant small molecule using mature calibration models; their regulatory success does not validate the biology or analytical performance of microneedle neuroprotein monitoring.

## 8. Compartment-Aware Interpretation Framework

The measured concentration is a function of cellular release, transport between compartments, peripheral handling, local skin biology, device recovery, and assay response. These processes may act simultaneously; multiplying them as fixed constants would misrepresent the underlying physiology.

In particular, the concentration measured in microneedle eluate should be distinguished from both the true local dISF concentration and the contemporaneous plasma concentration.

Relationships among CSF, blood, and dISF are dynamic across disease states, injury severities, time points, and devices [13,84]. Neurofluid transport, barrier integrity, renal and hepatic clearance, local skin physiology, and collection recovery can all contribute to cross-compartment discordance [26,117,180,187]. Minimum reporting should therefore include matrix, anatomical site, timing, device architecture and wear time, recovered-volume or normalization approach, assay platform, limits of quantification, and relevant physiological context.

## 9. Knowledge Gaps and Future Directions

The key gap is now clearer than a general call for more biomarker studies: proteins measured in plasma after brain injury have rarely been followed in the same people, at the same times, through a quantified dermal-ISF collection procedure. That missing linkage determines what a skin measurement can mean.

### 9.1. Biological and Analytical Knowledge Gaps

Existing paired designs show different pieces of the problem. Microdialysis and CSF were compared longitudinally in seven patients with severe TBI, with analyte- and patient-specific trajectories [34]. Paired plasma–dISF investigations in healthy volunteers have measured broad molecular and N-glycan overlap [27,167], while a small human protein study found variable serum–dISF relationships [111]. A wearable human aptamer patch followed vancomycin in dISF and required compartmental modelling to compare its time course with blood [174]. None of these studies simultaneously measured an established neurological injury protein in serial CSF, plasma, and dISF from patients after brain injury. A feasible first study would pair serial plasma and dISF from patients during the first day and the following week; add CSF only where already clinically indicated; and estimate analyte-specific recovery, lag, within-person variability, and the effect of sampling site. This would test whether dISF tracks plasma, integrates it over the wear interval, or chiefly reflects local skin biology.

Modelling can help specify these experiments. Atomistic simulations of stratum corneum lipids describe its structure and barrier properties, while models of the BBB endothelial membrane examine permeation and the free-energy barriers for selected small solutes [188,189,190]. These are useful mechanistic precedents, but they do not predict the passage of large circulating GFAP, NfL, or tau species through dermal capillaries or their recovery by a microneedle. Quantitative compartment models should therefore be fitted to paired human protein measurements and checked against independently measured device recovery. Permeability estimates from in silico, in vitro, and in vivo methods need matched definitions and careful benchmarking before they can be combined [191].

Another major knowledge gap is the extent to which glymphatic dysfunction alters peripheral biomarker kinetics. Brain-ISF Aβ and tau are demonstrably influenced by sleep–wake state [69,70], and glymphatic transport efficiency varies with circadian phase, age, and pathological condition [10,192,193], yet the extent to which these factors influence biomarker appearance in dISF remains poorly characterized. In military populations, this question may be further complicated by chronic sleep disruption and the high prevalence of blast-related mTBI. Piantino et al. [194] and Yamamoto et al. [195] demonstrated that veterans with blast-mTBI exhibit increased MRI-visible perivascular-space burden, a finding associated with altered glymphatic and interstitial-fluid clearance dynamics. However, the extent to which these physiological changes modify biomarker timing, concentration, or inter-compartment relationships remains unknown. Emerging non-invasive surrogates, including the DTI-ALPS index, perivascular-space burden on MRI, dynamic contrast-enhanced MRI, and magnetization-transfer spin labelling, may provide feasible approaches for investigating these relationships in future biomarker studies [196,197].

A further unresolved issue is the extent to which currently available assays measure comparable tau species across biological compartments. The existence of multiple tau proteoforms, including t-tau, p-tau181, p-tau217, p-tau231, BD-tau, and mass spectrometry-defined tau fragments, combined with the epitope dependency of immunoassay measurements, creates major cross-study incomparability that is compounded when novel matrices such as dermal ISF are introduced. SNTF is a separate calpain-cleaved spectrin fragment that may be considered as an axonal injury biomarker. Mass spectrometry-based proteoform mapping of tau across simultaneously collected CSF, plasma, and dermal ISF samples is therefore needed to determine which species are present in each compartment, at what concentrations, and with what kinetic profiles [19,136]. Similar unresolved questions apply to GFAP proteoforms, UCH-L1 degradation products, and the spectrum of NfL phosphorylation states.

Methodological standardization also remains limited. Consensus reporting standards for microneedle dISF biomarker studies equivalent to mature molecular-assay frameworks have not yet been established [26,117]. At minimum, reports should describe device geometry and material, anatomical site, insertion and wear conditions, skin preparation and tolerability, recovery and elution, volume or normalization method, analytical validation, storage, missingness, and paired-matrix timing.

### 9.2. Wearable Neuroproteomic Monitoring Platforms

Recent advances in microneedle sampling, ultrasensitive protein detection, and continuous sensing suggest that longitudinal dISF neuroproteomics may eventually be feasible outside conventional laboratories. Human MN systems have already demonstrated continuous measurement of metabolites and small molecules [172,173] but not continuous monitoring of low-abundance neuroproteins. Hydrogel-forming arrays are compatible with patch formats and temporal integration [117,155], while contemporary continuous-MN reviews define the engineering and calibration challenges that remain [198]. Active-reset aptamer systems and molecular-pendulum biosensors have enabled continuous protein monitoring in animal ISF [176,177]. Colorimetric p-tau181, electrochemical p-tau, and fluorescent MALAT1 platforms expand the available recognition and readout strategies [95,96,118]. The next useful experiment is a human study that measures recovered protein mass and paired plasma concentrations across the time window relevant to each analyte, alongside symptoms and other clinical measures.

If serial neuroprotein sensing becomes technically feasible, longitudinal interpretation will still require measured limits of detection, drift and fouling checks, within-person baselines, and prospective clinical validation. Wireless data transfer or automated analysis cannot compensate for an unknown blood-to-skin relationship [117,199].

### 9.3. Operational and Military Applications

Military and defence settings are a relevant application because blast-related mTBI and repetitive low-level blast exposure are difficult to assess objectively in the field [22,195]. Breachers may exhibit vestibular, executive, cortical, or inflammatory changes while remaining operationally active [200,201,202]. More recently, an integrated Bayesian analysis combining post-concussive symptoms, working-memory performance, and 13 circulating neuroproteomic markers found a 97% posterior probability of higher latent neuroinjury in experienced breachers than controls; symptoms contributed most strongly, biomarker contributions were smaller but consistent, and working memory did not load substantively [203]. MRI-visible perivascular-space burden raises additional questions about altered neurofluid transport after blast exposure [195]. A field-compatible, minimally invasive dISF patch could reduce phlebotomy and cold-chain demands if it were first shown to recover specific neuroinjury proteins reproducibly and to add actionable information beyond validated blood testing [204,205]. Continuous monitoring of GFAP, UCH-L1, NfL, or tau in human dISF should therefore be framed as a development objective, not as a current capability.

However, biomarker behaviour during operational exposure may be nonlinear. Tschiffely et al. [206] observed lower serum GFAP during intensive breacher training despite cumulative blast exposure, whereas other work reported transient post-blast elevations of UCH-L1 and spectrin breakdown products [207]. Repetitive subconcussive exposure may alter baselines, release patterns, compartment kinetics, or clearance in ways that cannot be inferred from single-impact clinical TBI.

Additional uncertainties concern performance under operational and physiological stressors. Exertion, dehydration, temperature extremes, sleep disruption, and variable skin conditions may alter dISF sampling or biomarker kinetics, yet these effects remain largely unstudied in operational populations. Translation from animal sensors also requires caution: Rubio et al. [208] identified biomechanical scaling differences between rodent and human blast exposure, and Ravin et al. [209] demonstrated species- and model-relevant astrocytic responses to simulated blast. Analyte-specific validation under realistic field conditions, human-factor assessment, and early engagement with the FDA, Health Canada, EMA (European Medicines Agency), and military procurement pathways will be needed before operational interpretation.

## 10. Conclusions

The concentration of a neurological injury protein in blood or dISF reflects its cellular source and molecular form, but also how it moves through CNS and peripheral compartments and how it is sampled. Glymphatic and meningeal lymphatic routes, barrier passage, dilution, and clearance may all contribute, with no established fixed contribution for each analyte [7,8,9,13]. An isolated peripheral result should therefore be interpreted against injury timing, assay characteristics, and the sampled matrix, rather than assumed to reproduce a brain or CSF concentration.

dISF is a credible source of systemic molecular information and a promising matrix for low-burden serial sampling. Human studies show broad metabolomic and proteomic overlap with plasma and support multiple collection workflows, while paired cytokine, metabolomic, and glycomic evidence also demonstrates analyte-specific concentration differences and local-tissue signals [27,54,111,140,141,142,143,144,167]. Microneedle and wearable platforms can detect systemic proteins, metabolites, p-tau species, and other neurodegeneration-oriented targets at human-feasibility, analytical, or preclinical stages [95,96,164,172,173]. A recent human aptamer-patch trial strengthens the case that dISF can support high-frequency molecular monitoring [174]; however, published evidence does not yet establish paired clinical dISF detection and concordance for GFAP, UCH-L1, NfL, tau, or Aβ. dISF must therefore not be treated as interchangeable with plasma or CSF.

Clinical translation requires synchronized multi-compartment time courses, proteoform-specific analytical validation, standardized device and reporting methods, and longitudinal testing in the intended population. The mechanistic case for investigating dISF is stronger than the present human neuroprotein evidence base. The appropriate conclusion is therefore cautious but constructive: microneedle-enabled dISF monitoring is a plausible minimally invasive complement to blood testing, provided that future studies demonstrate analyte-specific recovery, temporal linkage, biological validity, and clinical or operational utility.

## Figures and Tables

**Table 1:** Compartment-aware interpretation of neuroinjury biomarkers, including representative molecular mass, physiological confounds, and the present status of dermal-ISF measurement.

Biomarker	Primary CNS Source/Meaning	Brain ISF/Microdialysis	CSF	Blood (Plasma/Serum)	Dermal ISF via MN	Key Confounds/Notes
GFAP (~50 kDa)	Astroglial injury/reactivity	Early rise near lesion; local peaks; spatial heterogeneity [28]; Gfap was highly regulated in paired mouse ISF/CSF proteomics [35]	Robust proximal signal; affected by sampling site, turnover, and injury context	Detectable within hours of injury and may remain in blood for up to 7 days in TBI; FDA-cleared test [52]	High-priority candidate for paired validation; human dISF concordance not yet established	Interpret with time-from-injury; extracranial signal generally low
UCH-L1 (~25 kDa)	Neuronal cell-body injury	Rapid local increase; may be transient	Early CSF rise in acute injury	Earliest peripheral signal (1 h); sensitive to sampling window [52]	Candidate requiring early, short-cadence sampling; human dISF concordance not yet established	Consider polytrauma; hemolysis artefact; assay LOQ critical for mild TBI [21]
NfL (~68 kDa)	Axonal injury (delayed/persistent)	Elevates with axonal degeneration; slower dynamics	Delayed and sustained after acute ischemic stroke; associated with injury burden and outcome [92]	Excellent longitudinal monitoring candidate; post-injury signal may persist for weeks [89]	Promising for serial validation because persistent kinetics may tolerate integration; not yet clinically demonstrated in dISF	Influenced by age/comorbidities; interpret within-subject trajectories
Tau (t-tau, p-tau, BD-tau) (~45–65 kDa; forms vary)	Axonal/cortical injury; PTM/fragment-specific	Human microdialysis detects t-tau and p-tau181 and shows early extracellular tau dynamics after TBI and in neurosurgical patients [41,42,43]	Reflects CNS tau biology; total tau and p-tau can be measured in CSF, but interpretation depends on proteoform and disease context [19,44]	Detectability depends on assay/epitope; plasma p-tau181/217 shows strong diagnostic performance for AD [93,94]	Analytical and preclinical MN sensing reported for selected p-tau species [95,96]; no paired clinical dISF validation	Must specify analyte; avoid mixing tau species; glymphatic dysfunction affects kinetics [19]
NSE (~78 kDa dimer)	Neuronal enzyme; major extracranial sources (RBCs, platelets)	Can rise with injury	Used in post-cardiac arrest neuroprognostication [97]	Confounded by hemolysis and extracranial sources [98]	Lower priority unless hemolysis controlled	Use hemolysis index; interpret cautiously in extracranial injury [98,99]
S100B (~21 kDa dimer)	Astroglial; significant extracranial expression (adipose, muscle, melanocytes)	May rise but not specific to the CNS	Proximal signal; measurable in CSF, with established normative concentrations [100]	Confounded by exercise and polytrauma [68]	S100B detected preclinically in dISF from melanoma-expressing mice [101]; this demonstrates accessibility but also cutaneous-source confounding	Useful cautionary example of extracranial confound; early window only
Aβ42/40 ratio (~4.3–4.5 kDa)	Amyloid metabolism; vascular/clearance-sensitive	Serial brain-ISF Aβ reflects production, half-life, neuronal state, and injury physiology [39,40,41]	CSF Aβ42/40 ratio used in AD; influenced by clearance and compartment exchange [102]	Absolute concentrations are variable; Aβ42/40 ratios better reflect clearance biology [103]	Potential analytical target; clinical interpretation requires analyte-specific paired validation	Strongly influenced by glymphatic and vascular clearance dynamics
IL-6, TNF-α, VCAM-1, ICAM-1 (masses differ)	Neuroinflammation/vascular activation	Detectable with multiplex methods in microdialysate [28,33]	Reflects neuroinflammation + systemic spillover	Systemic confounds but essential modifier information [32]	High-plex PEA/NGS useful for modifier modelling [104]	Interpret as context covariates, not CNS-specific injury markers

Abbreviations: BD-tau: brain-derived tau; GFAP: glial fibrillary acidic protein; LOQ: limit of quantification; MN: microneedle; NfL: neurofilament light chain; NSE: neuron-specific enolase; p-tau: phosphorylated tau; PTM: post-translational modification; t-tau: total tau; UCH-L1: ubiquitin C-terminal hydrolase-L1. VCAM-1: vascular cell adhesion molecule-1; ICAM-1, intercellular adhesion molecule-1; PEA, proximity extension assay; NGS, next-generation sequencing; IL-6: Interleukin-6; TNF-α: Tumor Necrosis Factor-alpha; FDA: U.S. Food and Drug Administration. Approximate molecular masses identify commonly discussed intact monomers unless otherwise stated; circulating fragments, dimers, glycosylation, and assay epitopes change the effective hydrodynamic size. Protein isoelectric points are form- and milieu-dependent and have not been validated for the assay-defined species across these matrices. Intrinsic human systemic half-lives and blood-to-dISF lag times have not been established for these analytes. Reported blood windows represent apparent post-injury trajectories and should not be used as fixed clearance or skin-transfer parameters.

**Table 2:** Sampling and assay trade-space across compartments: practical considerations for neuroinjury biomarker monitoring.

Matrix	Invasiveness/Feasibility	Biological Proximity	Key Strengths	Key Limitations
Brain ISF (microdialysis/membrane-free sampling)	High; conventional human use is limited to neurocritical care/research and requires a burr hole; advanced proteomic/EV methods remain preclinical	Highest—local pericellular extracellular space; concentration depends on source proximity and probe recovery	Time-resolved mechanistic anchor; captures local chemistry; supports metabolic and protein profiling [28,33]; membrane-free methods enable rapid, region-resolved proteomics, and specialized protocols can recover brain-ISF extracellular vesicles [36,37,38]	Spatially local; recovery and analyte bias depend on method, probe geometry, membrane or pore size when used, flow, adsorption, and tissue response [29]; infection/hematoma risk; limited scalability
CSF (LP/EVD)	Moderate–high; clinical/research setting; LP or neurosurgical drain	High—proximal integrated signal; gradients relative to plasma are analyte-, site-, and barrier-dependent	Established for tau/Aβ AD diagnostics [102]; QAlb supports interpretation of CSF flow and blood–CSF barrier function [46]	Invasive; post-LP headache risk (~30%); contraindicated with raised ICP or coagulopathy; limited serial sampling; glymphatic and turnover effects on composition [137]
Blood (plasma/serum)	Low; highly scalable; venipuncture	Moderate—distal; shaped by glymphatic transport + BBB state + peripheral clearance	Operational feasibility; Established clinical evidence for blood GFAP/UCH-L1 testing; large normative datasets; NfL, GFAP, and p-tau clinically validated [52,89,93,108,115]	Large dilution gradient vs. CSF; peripheral clearance and binding confounds; BBB-state-dependent; hemolysis affects NSE [21,22]
Dermal ISF (microneedles)	Minimally invasive; scalable; potentially suitable for community, point-of-care, or wearable use [25,116]	Peripheral tissue matrix dynamically linked to plasma; no universal dISF–plasma ratio	Repeatable, low-burden sampling; no clotting; human proteomic, metabolomic, systemic-protein, and glycan measurements demonstrated [27,111,138,139,140,141,142]	Small or device-dependent recovered volume; normalization and local-tissue confounds; analyte-specific lag and recovery; no paired clinical validation for established neuroinjury proteins [143,144]
Suction blister ISF	Low–moderate; clinical/research setting	Moderate—plasma-equilibrated interstitial fluid	Larger volumes (50–200 µL); broad proteomic profiling possible; ACD and ICD can be discriminated by ISF proteomics + scRNA-seq [145]	Slow (30–90 min); equipment required; local inflammatory response from suction trauma; single time point only

Abbreviations: EVD: external ventricular drain; EV: extracellular vesicle; ICP: intracranial pressure; ISF: interstitial fluid; LP: lumbar puncture; MN: microneedle; POC: point of care; QAlb: CSF/serum albumin quotient; ACD: Allergic Contact Dermatitis; ICD: Irritant Contact Dermatitis; scRNA-seq: Single-cell RNA sequencing.

## Data Availability

No new data were created or analyzed in this study. Data sharing is not applicable to this article.

## Abbreviations

Aβ, amyloid-beta; AD, Alzheimer’s disease; ALS, amyotrophic lateral sclerosis; ApoE, apolipoprotein E; AQP4, aquaporin-4; BBB, blood–brain barrier; BD-tau, brain-derived tau; BCSFB, blood–cerebrospinal fluid barrier; BDP, breakdown product; CAA, cerebral amyloid angiopathy; CNS, central nervous system; CTE, chronic traumatic encephalopathy; CSF, cerebrospinal fluid; CV, coefficient of variation; DAI, diffuse axonal injury; dISF, dermal interstitial fluid; DTI-ALPS, diffusion tensor imaging analysis along the perivascular space; ECS, extracellular space; ePVS, enlarged perivascular space; EVs, extracellular vesicles; FTD, frontotemporal dementia; GFAP, glial fibrillary acidic protein; GCS, Glasgow Coma Scale; HIE, hypoxic–ischemic encephalopathy; HHPM, human health and performance monitoring; ICAM-1, intercellular adhesion molecule-1; ICP, intracranial pressure; IL, interleukin; ISF, interstitial fluid; IP-MS, immunoprecipitation mass spectrometry; lncRNA, long non-coding RNA; LLOQ, lower limit of quantification; LOD, limit of detection; LOQ, limit of quantification; MCI, mild cognitive impairment; MD, microdialysis; MN, microneedle; MRI, magnetic resonance imaging; mTBI, mild traumatic brain injury; NfH, neurofilament heavy chain; NfL, neurofilament light chain; NGS, next-generation sequencing; NPX, normalized protein expression; NSE, neuron-specific enolase; PD, Parkinson’s disease; PEA, proximity extension assay; p-tau, phosphorylated tau; PTM, post-translational modification; PVS, perivascular space; QAlb, CSF/serum albumin quotient; QIgG, CSF/serum immunoglobulin G quotient; RES, reticuloendothelial system; Simoa, single molecule array; SNTF, calpain-cleaved alpha-II spectrin N-terminal fragment; TBI, traumatic brain injury; t-tau, total tau; UCH-L1, ubiquitin C-terminal hydrolase-L1; VCAM-1, vascular cell adhesion molecule-1; VEGF, vascular endothelial growth factor.
